# Exploring local immunological adaptation of two stickleback ecotypes by experimental infection and transcriptome-wide digital gene expression analysis

**DOI:** 10.1111/j.1365-294X.2012.05756.x

**Published:** 2012-09-13

**Authors:** Tobias L Lenz, Christophe Eizaguirre, Björn Rotter, Martin Kalbe, Manfred Milinski

**Affiliations:** * Department of Evolutionary Ecology, Max Planck Institute for Evolutionary BiologyAugust-Thienemann-Str. 2, 24306, Plön, Germany; † Department of Evolutionary Ecology of Marine Fishes, GEOMAR, Helmholtz Centre for Ocean ResearchDüsternbrooker Weg 20, 24105 Kiel, Germany; ‡ GenXPro, Altenhöferallee 360438, Frankfurt am Main, Germany

**Keywords:** DGE, EST, *Gasterosteus aculeatus*, local adaptation, three-spined stickleback, transcriptome sequencing

## Abstract

Understanding the extent of local adaptation in natural populations and the mechanisms that allow individuals to adapt to their native environment is a major avenue in molecular ecology research. Evidence for the frequent occurrence of diverging ecotypes in species that inhabit multiple ecological habitats is accumulating, but experimental approaches to understanding the biological pathways as well as the underlying genetic mechanisms are still rare. Parasites are invoked as one of the major selective forces driving evolution and are themselves dependent on the ecological conditions in a given habitat. Immunological adaptation to local parasite communities is therefore expected to be a key component of local adaptation in natural populations. Here, we use next-generation sequencing technology to compare the transcriptome-wide response of experimentally infected three-spined sticklebacks from a lake and a river population, which are known to evolve under selection by distinct parasite communities. By comparing overall gene expression levels as well as the activation of functional pathways in response to parasite exposure, we identified potential differences between the two stickleback populations at several levels. Our results suggest locally adapted patterns of gene regulation in response to parasite exposure, which may reflect different local optima in the trade-off between the benefits and the disadvantages of mounting an immune response because of quantitative differences of the local parasite communities.

## Introduction

The need for individuals to survive and reproduce successfully in a given environment is the ultimate driver of evolution. Understanding the origin and extent of local adaptation among individuals is therefore a key objective of evolutionary biology and ecology (Greischar & Koskella 2007; Hereford 2009). Locally adapted individuals are expected to have higher fitness in their local environment in comparison with individuals from a different population and environment (Kawecki & Ebert 2004). Such patterns of higher fitness in the native environment are indeed supported by a growing number of studies from an increasing range of species, indicating that local adaptation is a frequent phenomenon in natural populations (Greischar & Koskella 2007; Hereford 2009; Fraser *et al*. 2011). However, despite this accumulating evidence at the individual level, the underlying physiological pathways and genetic mechanisms of local adaptation are often unknown (Bernatchez *et al*. 2010; Fraser *et al*. 2011).

Progress in this direction has been achieved by recent studies, using two different approaches. Experimental, hypothesis-driven candidate gene approaches have investigated the advantage of locally adapted genotypes, for example at the immunologically important major histocompatibility complex (Eizaguirre *et al*. 2012a) or at genes in involved in heat stress responses (Schwartz & Bronikowski This Issue), and provided valuable insights into the selection pressures leading to local adaptation. Other studies have taken a different route and employed exploratory genome-wide screens to identify genes with signatures of divergent selection (e.g. Elmer *et al*. 2010; Renaut *et al*. 2010; Roesti *et al*. 2012; Bourret *et al*. This Issue; Bruneaux *et al*. This Issue) or differential expression (e.g. Nolte *et al*. 2009; Goetz *et al*. 2010; Jeukens *et al*. 2010) between distinct populations or ecotypes. Such exploratory studies often pave the way for subsequent candidate gene approaches that allow for a more detailed investigation of the underlying selection pressures and mechanisms of local adaptation (Bernatchez *et al*. 2010). The latter approaches have also been tremendously facilitated by the onset of next-generation sequencing technologies and have improved our understanding of the genetic basis of local adaptation and population differentiation on a broader scale (Tautz *et al*. 2010). With these new technologies becoming more and more affordable, they offer an exciting and unprecedented opportunity for evolutionary ecology research of model and nonmodel species (Ellegren 2008; Ekblom & Galindo 2011).

Here, we make use of this new technological development, but go beyond the exploratory screen for differences between populations. By combining a hypothesis-driven experimental approach with the advantages of an unbiased genome-wide assessment of differential gene expression, we aim at improving our understanding of the genetic basis of local immunological adaptation in vertebrate populations.

The immune response of hosts is expected to be under strong selection, as parasites represent one of the major selective forces in evolution (Haldane 1992; Milinski 2006). Differences in abiotic and biotic conditions between different habitats are likely to influence the presence and abundance of potential intermediate hosts as well as parasites. Such differences may lead to contrasting and habitat-specific parasite communities (Thompson 1994; Kaltz & Shykoff 1998), which would in turn select for contrasting local immunogenetic adaptation of hosts that inhabit these different habitats (Dionne 2009; Eizaguirre & Lenz 2010). The adaptation to local parasite communities is therefore an intriguing and important aspect of local adaptation in natural populations. However, the investigation into differential immunological adaptation requires detailed knowledge of the study populations and their selective environment, that is, the predominating parasites.

The three-spined stickleback (*Gasterosteus aculeatus*) represents an ideal study system to investigate local adaptation. It inhabits marine as well as several different freshwater habitats and is well known for its adaptive radiations across the northern hemisphere (Bell & Foster 1994; Schluter & Conte 2009). Sticklebacks in northern Germany form genetically distinct populations between lake and river habitats, which are frequently referred to as ecotypes (Reusch *et al*. 2001; Eizaguirre *et al*. 2011). Such reduced gene flow between parapatric lake and river populations is also found for sticklebacks in northern America, indicating a repeated pattern of local adaptation to these different freshwater habitats (Berner *et al*. 2009; Roesti *et al*. 2012). While a number of different ecological factors seem to affect population divergence in sticklebacks (Kaeuffer *et al*. 2012), lake and river sticklebacks have been shown to differ in their resistance to parasites (Kalbe & Kurtz 2006; Scharsack *et al*. 2007). In fact, the parasite communities of these two freshwater habitats differ quantitatively and qualitatively in their species composition (Kalbe *et al*. 2002; Eizaguirre *et al*. 2011), and it has therefore been suggested that parasites play a significant role in the selection for local adaptation of lake and river sticklebacks. This hypothesis has already been proven for candidate genes of the major histocompatibility complex (MHC), which play a key role in the adaptive immune response (Eizaguirre *et al*. 2011, 2012a), but the genetic basis for potential local immunological adaptation beyond the MHC genes is still elusive.

While stickleback populations seem to harbour extensive standing genetic variation (Feulner *et al*. This Issue), it has also been shown that gene regulation provides a powerful repertoire for adaptation to new and local environments (Jones *et al*. 2012). To better understand the extent of immunological local adaptation in lake and river populations, we therefore exposed laboratory-bred three-spined sticklebacks to three common stickleback parasites and measured the hosts’ transcriptome-wide regulation of gene expression. This approach aimed at an unbiased estimate of which biological processes are activated in response to the parasite exposure. We hypothesize that lake sticklebacks, which are naturally exposed to a broader diversity of parasite species, have evolved a more potent general immune response, both at the innate and at the adaptive level, while river sticklebacks, which are naturally exposed only to restricted range of predominating parasites, may require more specific immune recognition (Eizaguirre *et al*. 2011, 2012a).

The goals of this study are twofold: Our main aim is to explore immune system–related transcriptional differences between lake and river sticklebacks, which may have evolved because of the well-characterized ecological differences between these two habitats and which could help explaining the previously observed difference in immunocompetence between these populations. Our second goal is to test the suitability of short read sequencing for genome-wide transcriptional profiling in molecular ecological research. For this, we employed digital gene expression analysis based on the SuperSAGE technique, which captures 26-bp-long sequence tags from the 3′-UTR of each gene transcript, originating from a specific restriction site and thus being identical for each transcript of a specific gene (Matsumura *et al*. 2005). As this approach does not require any normalization for transcript length in contrast to RNAseq, it allows for a more direct quantification of the underlying transcripts (Soanes *et al*. 2012). It is furthermore unbiased with respect to the sequences that can be detected, in contrast to the usual microarray approaches (‘t Hoen *et al*. 2008). In combination with next-generation sequencing, this technique represents a reliable and highly specific method for transcriptome-wide measurements of expression levels at an unprecedented scale (Matsumura *et al*. 2010; Sharbel *et al*. 2010). The specificity of the 26-bp tags per transcript was assured by the availability of the stickleback genome sequence (Jones *et al*. 2012) and further secured by sequencing a pool of full-length cDNA sequences from the experimental fish.

## Methods

### Experimental fish, parasite exposure and dissection

Three-spined sticklebacks from a lake (Großer Plöner See) and a connected river (Malenter Au) were naturally mated in the laboratory to produce pure lake and river families, respectively. Eggs were removed from the nest after fertilization and reared under standardized conditions in well water in aerated glass jars to minimize parental effects. Offspring of two lake and two river pairs, respectively, were raised in the laboratory under homogeneous conditions (18 °C, 16/24 h light, 10 individuals per tank) and a constant supply of frozen food. After five months, 16 randomly selected individuals of each of the four families were individually exposed to three macroparasites, two nematodes (*Anguillicoloides crassus*, *Camallanus lacustris*) and a digenean (*Diplostomum pseudospathaceum*), using established procedures (Wegner *et al*. 2003; Eizaguirre *et al*. 2012b). While *A. crassus* and *D. pseudospathaceum* occur in both original stickleback populations, *C. lacustris* is usually only found in lake sticklebacks (Eizaguirre *et al*. 2011). Exposure to the three parasites took place in individual 1-l tanks and separately on three consecutive days in the following order: Each fish received nine larvae each of *A. crassus* and *C. lacustris* (via infected copepods as intermediate hosts) and 20 larvae of *D. pseudospathaceum*. This dosage usually results in infection rate and intensity comparable to natural exposure (Eizaguirre *et al*. 2009b). 32 randomly selected additional fish of each family were not exposed but treated in the same way, including handling and supply with an equal number of naïve copepods. Fish were not fed on the day before the first exposure. After exposure, all fish were returned to larger tanks and kept in groups of eight individuals.

After 14 weeks, ten randomly selected individuals per family of the previously exposed fish were kept and exposed a second time to trigger activation of their adaptive immune system. Additionally, ten of the previously unexposed fish per family were randomly selected and now exposed for the first time. Individual exposure was again spread over three consecutive days, and each exposed fish received the same dose of each parasite larvae as before (for each of the two nematode parasites, larvae were transmitted via three infected copepods as intermediate hosts). The order in which fish were treated was randomized across all families to prevent any sequence effect between families. To serve as control, another ten of the previously unexposed fish per family were handled in the exact same way, except that their copepods did not contain any parasite larvae. While our digital analysis of gene expression was always aimed at sequencing only 24 individuals, the final number of 120 handled fish guaranteed enough backup to allow for random selection of the 24 individuals actually to be sequenced after all experimental procedures.

All 120 fish were dissected about 18 h after exposure to their last treatment in the same order as they had been handled the day before. This time point guaranteed activation of innate immunity, for example by the tissue-penetrating *D. pseudospathaceum* larvae, which take up to 24 h to reach their host's eye lenses (Rauch *et al*. 2006), as well as activation of potential memory cells of the adaptive immune system. Fish were sacrificed with an incision into the brain, because we feared that the stress of swimming in an anaesthetic could alter gene expression in the fish and thus jeopardize the whole experiment. All fish were handled in the same way with head–kidneys immediately stored in RNAlater for later RNA extraction. Gut, swim bladder and eyes were checked for *A. crassus*, *C. lacustris* and *D. pseudospathaceum* infection, respectively. All fish were dissected blind with respect to their treatment and origin, thus also unexposed fish were checked for parasites. All animal experiments described were approved by the Ministry of Agriculture, Environment and Rural areas in the State of Schleswig-Holstein, Germany.

### RNA library preparation and sequencing

RNA was extracted from head–kidneys using the NucleoSpin 96 RNA kit (Macherey-Nagel) according to the manufacturer's protocol, including rDNase treatment for removal of DNA. Head–kidneys are known to represent an immunologically very active organ (Press & Evensen 1999) and are commonly selected for immunological studies in teleosts. Resulting total RNA was subsequently stored at −80 °C, while an aliquot was kept for quality check on the 2100 Bioanalyzer (Agilent, USA). For individual SuperSAGE library preparation, we randomly selected RNA from one male and one female stickleback for each family and treatment among the samples with RIN value ≥ 7, leading to 24 individual libraries. SuperSAGE libraries were constructed by GenXPro GmbH (Frankfurt am Main, Germany) essentially as described by Matsumura *et al*. (2010) with slight modifications. Sequencing was performed on an Illumina GA II platform. For each library, 26-bp-long tags were extracted from the sequences using the GXP-Tag sorter software provided by GenXPro GmbH. Sequencing artefacts were reduced according to Akmaev & Wang (2004). To avoid bias during PCR of the tags, GenXPro's ‘TrueQuant’ method was employed: Each tag was individually barcoded by ligating artificial random 8-base pair oligonucleotides to the tags prior to the PCR. PCR copies were identified by analysing the combination of barcodes and tags, and similar combinations were eliminated from the data set.

As the published stickleback genome originates from a North American individual, it may exhibit certain sequence divergence from our European sticklebacks, especially in the evolutionarily less-constrained untranslated regions (UTR). To assure that the relatively short SuperSAGE tags would match successfully, we therefore decided to complement the sequence information from the genome with cDNA sequence information from our experimental fish. For whole transcriptome sequencing, we pooled RNA from the above selected individuals with additional RNA from other individuals of the same families and treatments in equal proportions. Poly(A)+ mRNA was isolated using a Poly(A)Purist mRNA purification kit according to the manufacturer's protocol (Ambion). The normalized, full-length-enriched cDNA library was generated using a SMART cDNA library construction kit (BD Clontech, USA) and a Trimmer Direct cDNA normalization kit (Evrogen, Russia), generally following the manufacturer's protocol but with several modifications. In brief, reverse transcription of 1 μg of poly(A)+ mRNA was performed with an Oligo-dT with a T7 polymerase binding site at its 5′-end. The resulting full-length-enriched, normalized cDNAs were linearly amplified by T7 RNA polymerase, resulting in a total of 10 μg RNA. The RNA was further processed for 454 and Illumina GAII sequencing according to the manufacturers’ protocols.

### Data filtering and bioinformatic processing

The raw sequences of the normalized full-length cDNA library were cleaned and filtered with the following tools: First, TagCleaner (Schmieder *et al*. 2010) was used to detect and trim sequencing adaptors at both read ends. Then the Galaxy pipeline (Blankenberg *et al*. 2001, 2010; Giardine *et al*. 2005; Goecks *et al*. 2010) was used for grooming of quality scores, for clipping of T7 adaptors, and for quality trimming by sliding window approach (window size of 5 bp and minimum aggregate quality score of 20). Finally, the PrinSeq pipeline (Schmieder & Edwards 2011) was used to filter the resulting sequences against less than 20 bp length, more than one N base, low complexity (maximum DUST score of 7), and exact read replicates. After filtering, the cDNA reads were used in a hybrid mapping assembly with the MIRA3 program under EST-optimized settings (Chevreux *et al*. 2004), which allows for mapping of reads from different technologies (here 454 and Illumina) against a backbone of reference transcripts and ranks among the most reliable transcriptome assembly tools (Mundry *et al*. 2012). Such a reference-guided mapping assembly of transcriptome reads has proven to be more successful than a de novo approach (Vijay *et al*. This Issue). As backbone sequences in MIRA, we used all 21 449 protein-coding stickleback transcripts with associated Gene Ontology ID, representing 16 371 genes, as available through ENSEMBL BioMart (full-length cDNA including UTR sequence, ENSEMBL version 65; Kinsella *et al*. 2011). The assembled sequences were then converted into a local BLAST database.

The individual SuperSAGE tags were filtered against the presence of ‘Ns’ and for the occurrence in at least 7 different libraries to avoid any bias in gene expression analysis by family-specific alleles (our SuperSAGE data set included 6 individuals per family). This very conservative approach ensures that detected differences between populations are not confounded by family-specific sequence variation. It also removes the vast number of low-frequency tags, which have no statistical power (Eveland *et al*. 2010). The remaining tags were mapped against the assembled transcripts using the BLASTn tool (Altschul *et al*. 1997). Tags were assigned to a given transcript if they matched uniquely (taking into account different splice variants per gene) in the correct orientation with less than two mismatches. Tag counts within each library were combined for genes with several transcripts (splice variants) and normalized to an overall library size of 1 million tags because of slight differences in the absolute number of tags per library (median: 2.3 million, range: 1.1–3.6 million), which would otherwise bias expression differences between individuals.

### Analyses of differential expression

All statistical analyses were performed in R (vers. 2.14.2, R Development Core Team 2012). A nonparametric, permutational multivariate analysis of variance (vegan package, Oksanen *et al*. 2012) based on the library-specific SuperSAGE tag counts per gene was performed to test for overall differences in the transcriptome-wide response to infection between the two populations (Zapala & Schork 2006). Multivariate analysis was based on a Pearson correlation distance matrix, calculated with the function Dist (amap package, Lucas 2011), which is suggested as a suitable metric for gene expression data sets (D'haeseleer 2005). The PerMANOVA was run with 999 permutations each on the whole data set and then separately for each treatment group (control, singly exposed, twice exposed). The models included treatment (if appropriate), population of origin, family background and sex.

We then employed a differential analysis of gene expression using the Bioconductor package edgeR (Robinson *et al*. 2010), which scored well in a recent comparison (Kvam *et al*. 2012), and allows for incorporating the experimental design (e.g. crossed treatment and population groups) during the estimation of gene-wise dispersion. Separately for both lake and river fish, we identified all genes that were significantly up- and down-regulated from control to once exposed fish and from once exposed to twice exposed fish. *P*-values were corrected for multiple testing according to Benjamini and Hochberg (FDR, Benjamini & Hochberg 1995).

Using information from the Gene Ontology (GO) database (Ashburner *et al*. 2000), we then identified functional associations of the up-regulated genes and tested whether they were enriched for particular biological processes. We focused on up-regulation of genes, because of its more straightforward interpretability. The GO annotations for each gene were obtained through ENSEMBL BioMart. Functional enrichment for biological processes (based on implemented Fisher's exact test) was calculated with the Bioconductor package topGO (Alexa & Rahnenfuhrer 2010). The gene pool against which to compare differentially expressed genes for the estimation of GO term enrichment was defined as the list of all genes against which we performed our BLAST search, that is, for which GO terms were available from ENSEMBL Biomart. Only GO terms supported by at least 5 different genes were included in the analysis to allow more thorough interpretation of the results. We compared functional enrichment in the expression response between populations both overall and specifically for immune system processes. The overall differences in GO term representation were visualized using the Bioconductor package goProfiles (Sanchez *et al*. 2010).

## Results

### Parasite load

The number of parasites per fish differed between parasite species, but was always significantly higher in twice exposed sticklebacks as would be expected (Mann–Whitney *U*-tests, all three *P* < 0.05; Table 1). Within exposure groups, river fish showed usually, but not always, higher parasite loads than lake fish. The most substantial difference in susceptibility between populations could be observed for *Diplostomum pseudospathaceum* (Table 1). Although in natural populations, *Camallanus lacustris* is only found in lake sticklebacks, we did not detect any differences in resistance/susceptibility to this parasite.

**Table 1:** Individual parasite load in lake and river fish after experimental exposure. Median number of parasite individuals (±SD) per stickleback is given for the three helminth parasites *Anguillicoloides crassus*, *Camallanus lacustris* and *Diplostomum pseudospathaceum,* respectively, which is used for experimental exposure of naïve lake and river sticklebacks. Each exposure population group represents 20 individuals (total exposed *N* = 80). *P*-values indicate significance of the differences in parasite load between lake and river fish (Mann–Whitney *U*-tests)

	*Anguillicoloides*		*Camallanus*		*Diplostomum*	
Median ± SD	*P*	Median ± SD	*P*	Median ± SD	*P*
Single exposure	Lake	0.0 ± 0.4	0.382	2.5 ± 3.1	0.150	1.5 ± 1.5	**<0.001**
	River	0.0 ± 0.7		1.0 ± 2.2		7.5 ± 4.4	
Double exposure	Lake	0.0 ± 0.5	**0.002**	6.5 ± 3.5	0.807	4.5 ± 2.5	**<0.001**
	River	1.0 ± 0.9	8.0 ± 3.6	21.5 ± 4.9

### Transcriptome mapping

After cleaning of sequences from the normalized cDNA pool, we obtained 171 146 454-reads with an average read length of 169 bp and 5.2 million Illumina reads with an average read length of 60 bp, resulting in a theoretical 9x coverage of the 21 449 ENSEMBL transcripts. The mapping assembly of our local cDNA sequences to the ENSEMBL transcripts introduced 10 835 IUPAC consensus bases, which is of critical importance for the subsequent BLAST-based approach. With the requirement of a unique hit and a maximum of one mismatch between SuperSAGE tag and transcript to score the respective gene, a segregating SNP of which one allele corresponds to the ENSEMBL sequence could lead to allele-specific instead of gene-specific estimates of gene expression. With the introduced consensus bases, each of the two alleles will have the same number of mismatches (the BLAST algorithm treats IUPAC bases as unknown Ns) and thus the same likelihood to score the underlying gene.

### SuperSAGE tags

After filtering for quality and minimal occurrence, we retained 85 301 unique SuperSAGE tags with an overall raw count of 51 508 850 across all libraries, of which 13 695 (44% of raw count) could successfully be associated with one of the ENSEMBL transcripts. It is expected for EST sequencing projects that the majority of the detected reads does not map to protein-coding genes and instead represent noncoding RNAs and other transcripts with unknown functions (Chen *et al*. 2002). Based on our BLAST call criteria, only a very small fraction (0.3%) of unique tags matched to transcripts of more than one gene, indicating that the 26 base pairs of the SuperSAGE tags are generally very specific and allow for reliable estimation of gene expression. Overall, we thus obtained expression data for 5709 of the 16 371 stickleback genes with GO annotation, with a median tag count per gene of 232 and a range from 7 to 762 800 (before normalization). The relatively low fraction of detected genes (34.8%) is presumably due to the specialized immunological function of the head–kidney, which is expected to only express a specialized subset of genes.

### Differential gene expression

**Figure 1:**
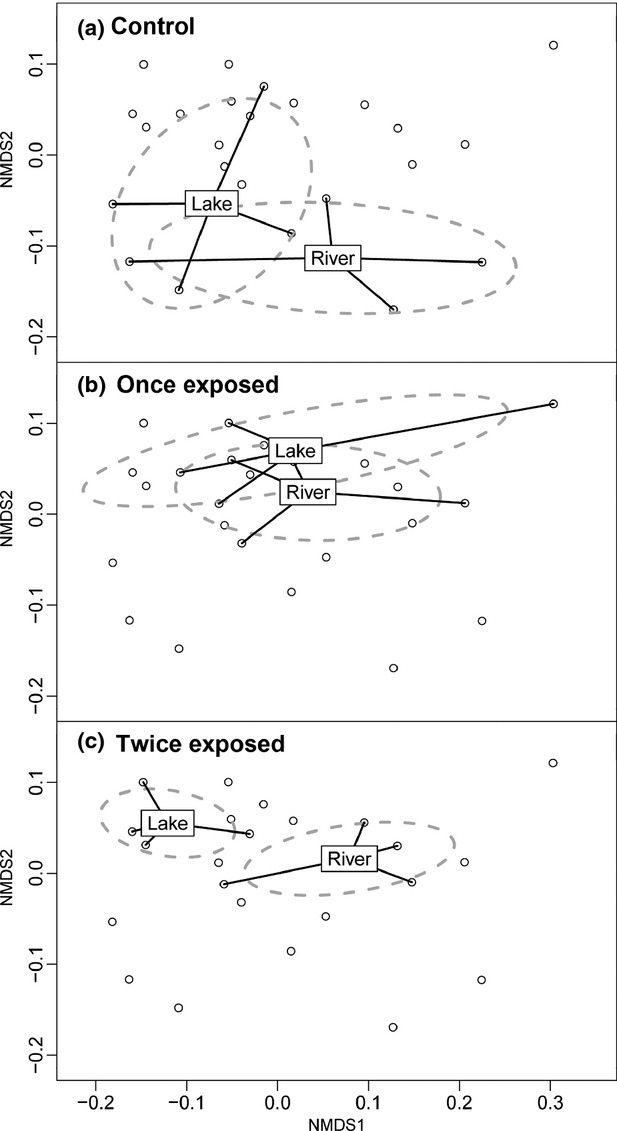
Multidimensional scaling plot of gene expression levels in lake and river fish. Multidimensional scaling (MDS) plots based on the tag counts per gene indicate similarity between individual samples (dots) in their expression pattern. Each treatment group is plotted separately (top to bottom: control, once exposed, twice exposed). The dashed circles indicate 95% confidence intervals for each population. NMDS: nonmetric multidimensional scaling score. Stress for two-dimensional representation: 0.10.

The multivariate analysis of variance showed that the overall expression levels across the three treatments (control, single exposure, double exposure) differed significantly between lake and river fish (Table 2). However, this effect was only visible in fish that had been exposed twice to activate their adaptive immune response (Table 2, Fig. 1c). Neither the unexposed control fish nor the once exposed fish showed such differences between populations (Table 2, Fig. 1a, b). This suggests that the two populations do not differ in general in their unstimulated gene expression, but that the exposure to parasites seems to trigger different expression responses in lake and river sticklebacks after multiple exposures. In addition to a difference in expression levels between singly exposed male and female fish, which may reflect sex-specific strategies of innate immunity, we also detected a trend towards different overall expression levels between families. With only two families per population, it is difficult to separate the effects of family-specific and population-specific gene expression. However, in twice exposed fish, where we find a significant difference between populations, we did not detect significant family differences, suggesting that the observed difference in gene activation can be attributed to population rather than family origin.

**Table 2:** Multivariate analysis of variance of transcriptome activation. The effect of experimental treatment (control, once exposed, twice exposed), population of origin (lake vs. river), family and sex on overall gene expression levels was tested with a permutational multivariate analysis of variance on a Pearson correlation distance matrix. Effects were tested on the whole data set (*N* = 24) and separately within the treatment groups (each *N* = 8). *F*-statistics (*F*), term-specific and residual degrees of freedom (d.f.) and *P*-values (*P*) are given for each model term

Factor	Overall			Control			Once exposed			Twice exposed		
*F*	d.f.	*P*	*F*	d.f.	*P*	*F*	d.f.	*P*	*F*	d.f.	*P*
Treatment	3.4	2/17	**0.046**	–	–	–	–	–	–	–	–	–
Population	6.5	1/17	**0.014**	6.6	1/3	0.078	0.1	1/3	0.752	11.9	1/3	**0.040**
Family	3.1	2/17	0.056	6.1	2/3	0.072	1.4	2/3	0.312	1.1	2/3	0.438
Sex	0.0	1/17	0.88	1.9	1/3	0.255	5.9	1/3	**0.037**	1.2	1/3	0.352

**Figure 2:**
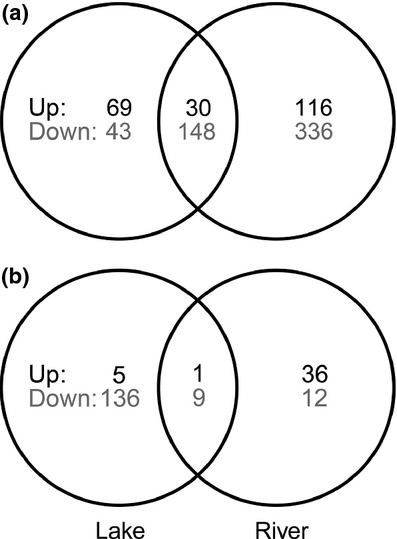
Differentially expressed genes between populations. The number of genes up- and down-regulated in fish of both or only of one of the populations. Expression changes from unexposed to once exposed (a) and from once exposed to twice exposed (b) fish are given.

When screening for genes with differential expression between treatments, we detected substantial changes in expression levels, which appeared to be mainly population specific (Fig 2, see supporting information for complete lists of differentially expressed genes). The most drastic changes occurred between unexposed control fish and once exposed fish, with most of the detected genes being down-regulated (Fig 2a). Interestingly, these regulatory changes were antagonistic between populations: While most of the genes with lake-specific differential expression were up-regulated upon single parasite exposure, most river-specific genes were down-regulated (Pearson's chi-squared test, χ^2^ = 53, *P* < 0.001). This pattern was inversed upon the second parasite exposure, which was hypothesized to activate the adaptive immune response. At this stage, most of the genes with lake-specific differential expression were down-regulated, while river fish appeared to activate more additional genes (χ^2^ = 106, *P* <0.001; Fig 2b).

**Figure 3:**
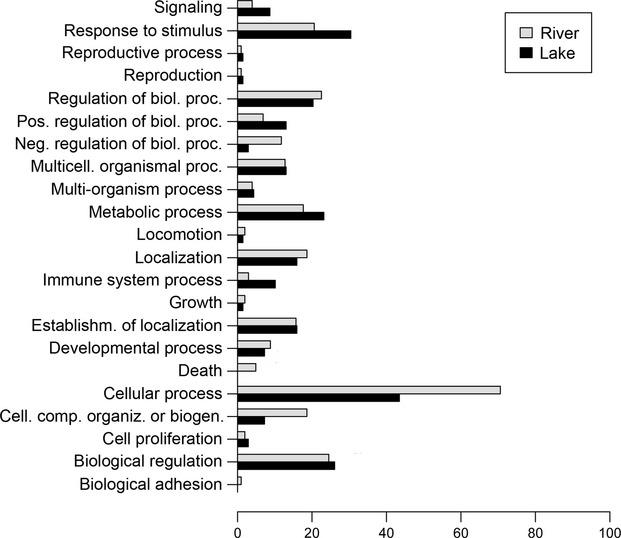
Up-regulated biological processes in lake and river fish. For each of the second-order GO terms, the proportion of population-specific up-regulated genes annotated with that term is shown for lake and river sticklebacks.

To understand the biological function of these genes and thus be able to interpret their activation, we investigated whether the population-specific up-regulated genes were enriched for roles in particular biological processes as defined by the Gene Ontology (GO) database. GO term enrichment analysis compares the fraction of observed genes that are annotated with a given GO term against the fraction of equally annotated genes in the whole set of genes of a given organism. This analysis works only for genes for which GO annotation of a given ontology category (here ‘Biological Processes’) is available. This reduced the number of genes for the GO term enrichment analysis from 16 371 to 11 184 genes with overall 8627 represented GO terms for biological processes. Among the population-specific up-regulated genes of lake and river fish, we found substantial differences in the representation of GO terms (Fig 3 and Table S1 & S2, Supporting information). While fish of both populations activated genes involved in the response to stimuli, lake fish increased expression of immune genes to a larger extent than river fish (Fig 3). River fish in contrast seemed to substantially activate cellular processes.

### Immune system processes

The difference in expression of immune genes was even more apparent when explicitly testing for the enrichment of genes involved in immune system processes. While immune system processes in general appeared to be up-regulated upon parasite exposure in all fish, this effect was mainly due to up-regulation in lake fish and could not be detected in river fish (Table 3). When differentiating between processes of the innate and the adaptive immune system, a significant enrichment could only be detected for the former. We also observed that up-regulation of immune system processes in lake fish occurred primarily upon first exposure. The number of genes additionally up-regulated in fish exposed a second time was substantially lower than from naïve to first exposure (Table 3), which leads to reduced power in detecting significant enrichment for specific GO processes. This is also reflected in the overall GO term enrichment analysis at this stage, with none of the tested GO terms surviving FDR correction (data therefore not shown). Eventually, we investigated also the genes that were up-regulated in fish of both populations simultaneously upon first parasite exposure, but found GO term enrichment only for metabolic and cellular processes (Table S3, Supporting information).

**Table 3:** Up-regulation of immune system processes in lake and river fish. The number (#) of up-regulated genes is given for each GO term, comparing overall expression changes from control to all exposed fish and population-specific changes from control to once exposed and once exposed to twice exposed fish. The *P*-values indicate whether these numbers are higher than expected from GO term associations in the global set of genes. The parentheses show the actual number of significantly up-regulated genes (overall or population specific)

GO term ID	GO term description	Control to all exposed		Control to once exposed				Once exposed to twice exposed			
Overall (232)		Lake (44)		River (77)		Lake (5)		River (23)	
#	*P*	#	*P*	#	*P*	#	*P*	#	*P*
GO:0002376	Immune system processes	11	**0.021**	9	**<0.001**	3	0.792	0	–	0	–
GO:0045087	Innate immune system	2	0.470	2	**0.036**	1	0.409	0	–	0	–
GO:0002250	Adaptive immune system	2	0.285	1	0.182	1	0.297	0	–	0	–

## Discussion

Combining the depth and unbiasedness of next-generation sequencing technology with the interpretational strength of a hypothesis-driven experimental approach, we were able to detect potential differences in the transcriptome-wide response to parasite exposure between families of two stickleback ecotypes, suggesting a significant extent of local immunological adaptation to the distinct parasite-mediated selection regimes in their respective habitats.

River sticklebacks appeared overall more susceptible to our experimental parasite exposure, which was most apparent for *Diplostomum pseudospathaceum*. The natural prevalence of this digenean parasite is substantially higher in lake populations, and lake sticklebacks have already been shown to exhibit higher immunocompetence against *Diplostomum* (Kalbe & Kurtz 2006). It has been argued that this effect is due to an elevated innate immune response, as the higher resistance shows up already upon first contact with this parasite, which is furthermore known to evade adaptive immunity by migrating to the host's eye lens within about a day after penetrating the host's skin (Kalbe & Kurtz 2006; Rauch *et al*. 2006).

Our digital analysis of gene expression on the basis of 5709 detected protein-coding genes showed significant differences between fish from the two populations, each represented by two families, suggesting that lake and river sticklebacks activate different pathways in response to our parasite exposure. This could be seen at the global expression level, using multivariate statistics, and also at the functional level by GO term enrichment analysis. The multivariate analysis also showed that the difference in expression levels was triggered by the parasite exposure and was not due to a general difference in gene regulation between lake and river sticklebacks, as no significant difference in global expression levels could be detected in unexposed fish. While overall, the sticklebacks appeared to activate their immune system upon our experimental parasite exposure as indicated by an enrichment for immune system processes, when focussing on population-specific gene regulation, this pattern could only be observed in lake fish. River fish did not show any particular activation of immune system processes, but instead of cellular catalytic processes. Furthermore, immune system activation in lake fish upon first exposure affected mainly processes of the innate immune system, which follows our expectations as it represents the first line of defence upon first contact to a parasite. Only a substantially smaller number of additional genes were altered upon second exposure, which is when an adaptive immune response would be expected. It may be possible that the relatively high number and infectivity of *Diplostomum* larvae in our experiment to a certain degree dominated the second parasite challenge and thus led to re-activation of the innate immune response. This in turn may render the detection of adaptive immune processes more difficult. On the other hand, the multivariate approach detected overall differences in gene expression between populations especially in the twice exposed fish. It may therefore be that the up-regulation of the adaptive processes is more subtle and was obscured in the GO term analysis by an additional re-activation of the innate immune system.

Because of the still inherent sample size limitation of next-generation sequencing techniques, we were able to screen two different families per stickleback population, which may limit the interpretation of our results. In addition to local adaptation, transcriptional differences may also partly be due to family effects, genetic drift or other random effects. However, previous experimental work has already established that lake sticklebacks generally exhibit a higher immunocompetence compared with river sticklebacks (Kalbe & Kurtz 2006; Scharsack *et al*. 2007), which potentially evolved in response to the significantly broader parasite diversity in lakes (Kalbe *et al*. 2002; Eizaguirre *et al*. 2011). We have also shown previously that the genes of the major histocompatibility complex (MHC), key components of the adaptive immune system, are locally adapted to this difference in parasite diversity and thus partly responsible for the differences in parasite resistance between lake and river sticklebacks (Eizaguirre *et al*. 2011, 2012a). In the light of these previous findings and the well-characterized genetic differentiation of these populations in general (Reusch *et al*. 2001; Eizaguirre *et al*. 2011), it is therefore justified to suggest that our discovery of an activation of different biological processes in response to parasite exposure between fish from the two populations could indicate population-specific differences in the regulation of the immune system, which may have evolved in adaptation to differences in their local parasite communities.

We can here only speculate as to whether a potential difference in immune system activation is an adaptation to qualitative differences between lake and river parasite communities or whether it is merely an adaptation to quantitative differences. However, the fact that the functional differences were mainly observable in pathways of the innate immune system may indicate the latter, and it is conceivable that lake sticklebacks have evolved mechanisms to activate a stronger innate immune response upon parasite exposure as a consequence of the stronger and more diverse parasite challenges in their native habitat. An evolutionary response to qualitative differences may indeed require more specific adaptations than the mere differential regulation of immune genes, for example the evolution of locally adapted MHC genotypes (Eizaguirre *et al*. 2012a), especially given the potential of parasites to co-evolve with their hosts (Ebert & Hamilton 1996).

River sticklebacks in turn may have evolved a more diminished activation of the immune system as a consequence of the reduced parasite challenge in their habitat, because especially the components of the rather unspecific innate immunity can be severely damaging. In fact, the immune response is expected to follow a fine-tuned trade-off between the benefits of pathogen resistance and the disadvantages of energetic costs and tissue damage (Schulenburg *et al*. 2009). Our data could thus indicate a difference in the location of the optimal trade-off between the two populations due to the quantitative differences in their parasite communities. Instead of triggering a broad innate immune response, river sticklebacks may rely on a more specific immune response, for instance facilitated by the antigen-presenting molecules of the MHC (Wegner *et al*. 2007; Eizaguirre *et al*. 2012a). The activation of cellular processes in river sticklebacks upon parasite exposure could either indicate a general response to stress (de Nadal *et al*. 2011) or an attempt to increase tolerance to the parasite infection (Medzhitov *et al*. 2012). The latter strategy, which has been proposed as an alternative to increasing resistance mechanisms, has for instance been raised in explaining the surprising lack of immune gene regulation in the transcriptomic response of parasite-challenged red grouses (*Lagopus lagopus scoticus*; Webster *et al*. 2011). Similarly, Pemberton *et al*. (2011) describe a surprising lack of differential expression in immune genes between control and infected Scottish blackface lambs (*Ovis aries*), further emphasizing the potential complexity of immune responses in general. An interesting addition to the trade-off scenario is the observed difference in gene expression levels between the sexes after the first exposure, which may reflect additional sex-specific optima for the activation of the rather unspecific and thus more costly innate immune system (Restif & Amos 2010).

Overall, our study highlights the suitability of digital gene expression analysis, based on the SuperSAGE protocol, for investigating immunological adaptation of diverging vertebrate populations and provides new insights into the mechanisms that may be involved in this process. With two families per population, we captured only a subset of the variation in gene expression that undoubtedly exists in each natural population, and some of the observed differences between populations may be confounded by family-specific gene expression (Ma & Qin 2007). Using F1 families, we can also not completely rule out parental effects. While we used a very conservative approach by removing family-specific sequence tags and furthermore did not detect family-specific expression differences in exposed fish, more data are needed to confirm that the observed patterns reflect population-specific local adaption of transcriptome regulation. It also remains to be investigated whether the observed differences are specific to the two study populations or represent a more general scenario for ecotypes that evolve under different parasite communities. In fact, it has already been suggested that regulatory changes provide a potent means for adaptation to new environments (Jones *et al*. 2012). Furthermore, in a recent comparison of general transcriptome-wide expression levels between two unchallenged morphs of lake trout (*Salvelinus namaycush)*, Goetz *et al*. (2010) have also found most differential expression in genes associated with metabolism and immunity, supporting the notion that local adaptation to parasite communities may be a common scenario among diverging populations, a process that may ultimately lead to the formation of distinct species (Eizaguirre *et al*. 2009a).

## 

T.L.L. is interested in immunogenetic local adaptation of vertebrates and the maintenance of genetic diversity through host-parasite coevolution. He uses field-based, experimental and computational approaches to study various species from fish to humans. C.E. is interested in host-parasite co-evolution and its link to mating strategies, speciation and conservation. B.R. is laboratory leader at GenXPro-GmbH and involved in the development of NGS techniques for analysis of gene expression, genotyping and epigenetics. His major research interest is currently to combine all nucleotide-based information of biologic systems in a larger context. M.K. is interested in ecological aspects of parasites and investigates local adaptation of sticklebacks and their natural worm parasites as a co-evolutionary snapshot, particularly by combining field surveys with infection experiments in the lab. M.M. is interested in the evolutionary ecology of the Major Histocompatibilty Complex (MHC) with respect to host-parasite co-evolution and host mate choice.

## Data accessibility

Lists of enriched GO terms and differentially expressed genes are available online as supplementary information. Expression and parasite data are available through the Dryad repository (Lenz *et al*. 2012): http://dx.doi.org/10.5061/dryad.478g5.

## Supporting Information

**Table S1.** Enriched GO terms in exposed lake sticklebacks. **Table S2.** Enriched GO terms in exposed river sticklebacks. **Table S3.** Overall enriched GO terms upon parasite exposure.

Data S1 Lists of significantly up- and down-regulated genes between treatments for each population.
