# Supplementary material for: Exploring local immunological adaptation of two stickleback ecotypes by experimental infection and transcriptome-wide digital gene expression analysis
Source: Mol Ecol. 2012 Sep 13;22(3):774–86. doi: 10.1111/j.1365-294X.2012.05756.x (PMC3579235; doi:10.1111/j.1365-294X.2012.05756.x)
Supplement: Supplementary file 1 — Table S1. Enriched GO terms in exposed lake sticklebacks. Table S2. Enriched GO terms in exposed river sticklebacks. Table S3. Overall enriched GO terms upon parasite exposure. [file mec0022-0774-sd1.pdf]

Supplementary table S1: Most enriched GO terms of biological processes among up-regulated genes with lake-specific differential expression between control and singly exposed sticklebacks

GO.ID	Term	Overall	Up-regulated	Expected	classicFisher	FDR
GO:0006508	proteolysis	731	22	4.51	2.00E-10	8.02E-07
GO:0006956	complement activation	7	4	0.04	4.60E-08	6.15E-05
GO:0072376	protein activation cascade	7	4	0.04	4.60E-08	6.15E-05
GO:0009308	amine metabolic process	327	12	2.02	6.30E-07	0.0006
GO:0009063	cellular amino acid catabolic process	34	5	0.21	1.90E-06	0.0015
GO:0009072	aromatic amino acid family metabolic pro...	16	4	0.1	2.30E-06	0.0015
GO:0006959	humoral immune response	18	4	0.11	3.80E-06	0.0021
GO:0006955	immune response	262	10	1.62	4.20E-06	0.0021
GO:0009310	amine catabolic process	42	5	0.26	5.50E-06	0.0024
GO:0016054	organic acid catabolic process	58	5	0.36	2.70E-05	0.0098
GO:0046395	carboxylic acid catabolic process	58	5	0.36	2.70E-05	0.0098
GO:0044106	cellular amine metabolic process	269	9	1.66	3.80E-05	0.0127
GO:0002252	immune effector process	110	6	0.68	5.70E-05	0.0172
GO:0002253	activation of immune response	68	5	0.42	6.00E-05	0.0172
GO:0006520	cellular amino acid metabolic process	229	8	1.41	7.80E-05	0.0208
GO:0050776	regulation of immune response	119	6	0.73	8.90E-05	0.0223
GO:0050778	positive regulation of immune response	81	5	0.5	0.0001	0.0330
GO:0006547	histidine metabolic process	5	2	0.03	0.0004	0.0706
GO:0006548	histidine catabolic process	5	2	0.03	0.0004	0.0706
GO:0009075	histidine family amino acid metabolic pro...	5	2	0.03	0.0004	0.0706
GO:0009077	histidine family amino acid catabolic pro...	5	2	0.03	0.0004	0.0706
GO:0006725	cellular aromatic compound metabolic pro...	105	5	0.65	0.0005	0.0838
GO:0019439	aromatic compound catabolic process	7	2	0.04	0.0008	0.1342
GO:0008152	metabolic process	7070	56	43.62	0.0010	0.1587
GO:0019538	protein metabolic process	2794	29	17.24	0.0014	0.2213
GO:0019752	carboxylic acid metabolic process	359	8	2.21	0.0016	0.2346
GO:0043436	oxoacid metabolic process	359	8	2.21	0.0016	0.2346
GO:0006082	organic acid metabolic process	363	8	2.24	0.0017	0.2420
GO:0002684	positive regulation of immune system pro...	142	5	0.88	0.0018	0.2488
GO:0042180	cellular ketone metabolic process	370	8	2.28	0.0019	0.2552

Supplementary table S2: Most enriched GO terms of biological processes among up-regulated genes with river-specific differential expression between control and singly exposed sticklebacks

GO.ID	Term	Overall	Up-regulated	Expected	classicFisher	FDR
GO:0044275	cellular carbohydrate catabolic process	70	7	0.48	4.70E-07	0.0013
GO:0046164	alcohol catabolic process	73	7	0.5	6.30E-07	0.0013
GO:0016052	carbohydrate catabolic process	90	7	0.62	2.60E-06	0.0035
GO:0006006	glucose metabolic process	94	7	0.65	3.50E-06	0.0035
GO:0006096	glycolysis	44	5	0.3	1.20E-05	0.0096
GO:0019318	hexose metabolic process	118	7	0.81	1.60E-05	0.0107
GO:0005996	monosaccharide metabolic process	131	7	0.9	3.10E-05	0.0144
GO:0044262	cellular carbohydrate metabolic process	296	10	2.04	3.30E-05	0.0144
GO:0006066	alcohol metabolic process	240	9	1.65	3.80E-05	0.0144
GO:0006007	glucose catabolic process	56	5	0.39	3.90E-05	0.0144
GO:0006091	generation of precursor metabolites and ...	137	7	0.94	4.20E-05	0.0144
GO:0019320	hexose catabolic process	57	5	0.39	4.30E-05	0.0144
GO:0046365	monosaccharide catabolic process	58	5	0.4	4.70E-05	0.0145
GO:0044282	small molecule catabolic process	533	13	3.67	6.30E-05	0.0180
GO:0046034	ATP metabolic process	98	5	0.67	0.0006	0.1478
GO:0006457	protein folding	99	5	0.68	0.0006	0.1478
GO:0043968	histone H2A acetylation	6	2	0.04	0.0007	0.1627
GO:0006936	muscle contraction	62	4	0.43	0.0009	0.1915
GO:0003012	muscle system process	66	4	0.45	0.0011	0.2279
GO:0051443	positive regulation of ubiquitin-protein...	8	2	0.06	0.0013	0.2566
GO:0005975	carbohydrate metabolic process	471	10	3.24	0.0014	0.2606
GO:0044281	small molecule metabolic process	3624	38	24.95	0.0014	0.2606
GO:0046174	polyol catabolic process	9	2	0.06	0.0016	0.2841
GO:0051351	positive regulation of ligase activity	10	2	0.07	0.0020	0.3391
GO:0046483	heterocycle metabolic process	689	12	4.74	0.0025	0.3977
GO:0009152	purine ribonucleotide biosynthetic proce...	87	4	0.6	0.0030	0.4626
GO:0009260	ribonucleotide biosynthetic process	91	4	0.63	0.0035	0.5241
GO:0009056	catabolic process	1026	15	7.06	0.0039	0.5512
GO:0043967	histone H4 acetylation	14	2	0.1	0.0040	0.5585
GO:0051438	regulation of ubiquitin-protein ligase a...	17	2	0.12	0.0060	0.7951

Supplementary table S3: Most enriched GO terms of biological processes among up-regulated genes with common differential expression between control and singly exposed sticklebacks

GO.ID	Term	Overall	Up-regulated	Expected	classicFisher	FDR
GO:0006508	proteolysis	731	13	1.63	8.90E-10	3.57E-06
GO:0006547	histidine metabolic process	5	2	0.01	4.80E-05	0.0385
GO:0006548	histidine catabolic process	5	2	0.01	4.80E-05	0.0385
GO:0009075	histidine family amino acid metabolic pr...	5	2	0.01	4.80E-05	0.0385
GO:0009077	histidine family amino acid catabolic pr...	5	2	0.01	4.80E-05	0.0385
GO:0006026	aminoglycan catabolic process	14	2	0.03	0.0004	0.2749
GO:0005975	carbohydrate metabolic process	471	6	1.05	0.0005	0.2749
GO:0000272	polysaccharide catabolic process	19	2	0.04	0.0008	0.4009
GO:0016052	carbohydrate catabolic process	90	3	0.2	0.0010	0.4544
GO:0008152	metabolic process	7070	23	15.8	0.0012	0.4891
GO:0044238	primary metabolic process	6068	21	13.56	0.0018	0.6633
GO:0009063	cellular amino acid catabolic process	34	2	0.08	0.0026	0.8619
GO:0019538	protein metabolic process	2794	13	6.25	0.0033	1.0000
GO:0009310	amine catabolic process	42	2	0.09	0.0039	1.0000
GO:0006022	aminoglycan metabolic process	48	2	0.11	0.0051	1.0000
GO:0009308	amine metabolic process	327	4	0.73	0.0056	1.0000
GO:0016054	organic acid catabolic process	58	2	0.13	0.0074	1.0000
GO:0046395	carboxylic acid catabolic process	58	2	0.13	0.0074	1.0000
GO:0006027	glycosaminoglycan catabolic process	5	1	0.01	0.0111	1.0000
GO:0043462	regulation of ATPase activity	5	1	0.01	0.0111	1.0000
GO:0005976	polysaccharide metabolic process	79	2	0.18	0.0133	1.0000
GO:0010043	response to zinc ion	6	1	0.01	0.0133	1.0000
GO:0015695	organic cation transport	7	1	0.02	0.0156	1.0000
GO:0006000	fructose metabolic process	9	1	0.02	0.0200	1.0000
GO:0006032	chitin catabolic process	9	1	0.02	0.0200	1.0000
GO:0046686	response to cadmium ion	10	1	0.02	0.0221	1.0000
GO:0019674	NAD metabolic process	11	1	0.02	0.0243	1.0000
GO:0006030	chitin metabolic process	12	1	0.03	0.0265	1.0000
GO:0043270	positive regulation of ion transport	18	1	0.04	0.0395	1.0000
GO:0042742	defense response to bacterium	23	1	0.05	0.0502	1.0000
